# Early Cyanobacteria and the Innovation of Microbial Sunscreens

**DOI:** 10.1128/mBio.01262-19

**Published:** 2019-06-11

**Authors:** Patrick M. Shih

**Affiliations:** a Department of Plant Biology, University of California, Davis, Davis, California, USA; b Genome Center, University of California, Davis, Davis, California, USA; c Feedstocks Division, Joint BioEnergy Institute, Emeryville, California, USA; d Environmental Genomics and Systems Biology Division, Lawrence Berkeley National Laboratory, Berkeley, California, USA

**Keywords:** molecular clock, photosynthesis, sunscreen

## Abstract

Metabolism drives life; thus, understanding how and when various branches of metabolism evolved provides a critical piece to understanding how life has integrated itself into the geochemical cycles of our planet over billions of years. Although the most transformative metabolisms that have significantly altered the trajectory of Earth are inherently linked to primary metabolism, natural products that stem from specialized metabolic pathways are also key components to many auxiliary facets of life. Cyanobacteria are primarily known as the original inventors of oxygenic photosynthesis, using sunlight to split water to create our dioxygen-filled atmosphere; however, many of them also have evolved to produce small molecules that function as sunscreens to protect themselves from ultraviolet radiation. Determining when cyanobacteria first evolved the ability to biosynthesize such compounds is an important piece to understanding the rise of oxygen and the eventual success of the phylum.

## Abstract (precis)

Metabolism drives life; thus, understanding how and when various branches of metabolism evolved provides a critical piece to understanding how life has integrated itself into the geochemical cycles of our planet over billions of years.

## COMMENTARY

Arguably the greatest transition in our planet’s history has been the rise of oxygen in our atmosphere ∼2.3 billion years ago (Ga [giga-annum]). It is widely accepted that this is due to the biological innovation of oxygenic photosynthesis in ancient cyanobacteria, and a significant body of work has focused on constraining the timing of this monumental evolutionary innovation (1–3). Although much emphasis has been devoted to studying the events leading up to the evolution of oxygenic photosynthesis, the subsequent accumulation of dioxygen dramatically broadened the scope of metabolism for life as a whole. For example, this enabled widespread aerobic respiration; moreover, biochemical reactions that require oxygen (e.g., cytochrome P450s, terpene cyclases, etc.) most likely did not evolve until after the accumulation of oxygen in our atmosphere. Studying how life responded to major environmental changes—in terms of evolving new metabolisms—provides an important dimension to investigating the impact of such geochemical transitions, and thus these second-order metabolic innovations provide important case studies that enrich our understanding of the intertwined “give-and-take” between life and its abiotic environment.

Specific to early cyanobacteria, the accumulation of dioxygen via oxygenic photosynthesis would have undoubtedly led to significant amounts of cellular oxidative stress (4). Specifically, cyanobacteria require light to grow autotrophically, but with that light comes UV radiation. Cyanobacteria most likely evolved from anoxygenic phototrophs; thus, ancient stem group cyanobacteria would have initially produced small amounts of dioxygen. Hence, it has been proposed that these first cyanobacteria would have had to find nonenzymatic means to mitigate oxidative stress from the small amounts of O_2_ being produced (4). Eventually, the biosynthesis of small molecules that could function as antioxidants and/or photoprotective sunscreens would have dramatically enabled the transition to survive the intracellular production of O_2_ from oxygenic photosynthesis and eventually the oxygenated planet.

**FIG 1:**
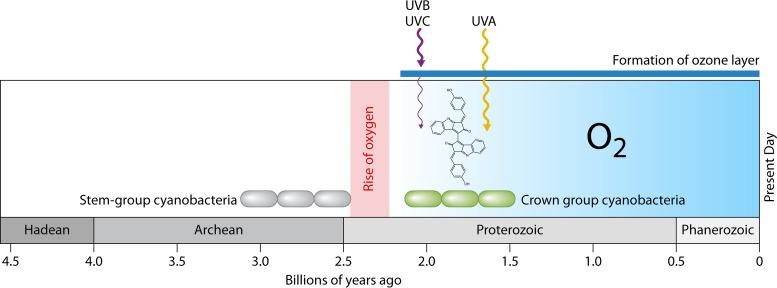
The rise of dioxygen in Earth’s atmosphere enabled various environmental transitions and biological innovations. Early stem group cyanobacteria may have been the progenitors of oxygenic phototrophs; however, those lineages would eventually go extinct. Adaptation to the changing environment, such as UV radiation, through the evolution of sunscreen biosynthesis in microbes would have enabled early phototrophs to adapt to specific environmental niches. The accumulation of oxygen ∼2.4 Ga enabled the formation of the ozone layer protecting against UVB and UVC radiation, but not UVA. Thus, there would have been selective pressure to evolve novel biosynthetic pathways to produce photoprotective sunscreens, such as scytonemin, in various lineages of crown group cyanobacteria.

Prior to the rise of oxygen in our atmosphere, there was no protective ozone layer, which absorbs UV radiation below 320 nm in wavelength (UVB and UVC). However, even after the formation of the ozone layer from the eventual accumulation of atmospheric oxygen, UV radiation between 320 and 400 nm (UVA) could still penetrate and mediate harmful photosensitized reactions mediating oxidative stress (Fig. 1). Thus, many cyanobacteria have a love-hate relationship with sunlight, where too much of a good thing can be bad. Like humans, cyanobacteria can utilize sunscreens to protect themselves from UV damage, albeit they have had to evolve specialized branches of metabolism to biosynthesize such compounds. Understanding how cyanobacteria evolved to cope with such oxidative stresses through the evolution of sunscreen biosynthetic pathways is an important part to understanding the early successes of cyanobacteria. Importantly, O_2_ derived from photosynthesis does not simply accumulate in the cell, as rates of respiration contribute to the consumption of dioxygen. Along these lines, intracellular concentrations of dioxygen in photosynthesizing cyanobacteria are lower than previously expected based on modeling studies; thus, investigating at what point there was an evolutionary advantage to biosynthesize sunscreens to protect cells from oxidative damage has been an open question (5). Garcia-Pichel et al. expand upon previous genetic/genomic work on elucidating the sunscreen biosynthetic gene cluster of scytonemin, a widespread cyanobacterial sunscreen, with molecular clock analyses to help constrain the timing of the cyanobacterial phylum and the evolution of scytonemin biosynthesis (6).

Garcia-Pichel et al. reconstruct the phylogeny and carry out molecular clock analyses of various enzymes conserved in the scytonemin operon. Because scytonemin is derived from various aromatic amino acids, a unique feature of the scytonemin operon is that several aromatic amino acid biosynthetic enzymes have been recruited following a gene duplication event. Thus, a second copy of core aromatic amino acid biosynthetic genes is found within the scytonemin operon, enabling the demarcation of core genes associated with scytonemin. In general, molecular clock analyses aim to utilize time constraints, such as fossils or geological constraints, to calibrate and introduce reference points to estimate when specific events across a phylogenetic tree occurred. Importantly, the selection and justification of markers for such analyses are essential to the credibility of the study; thus, many previous studies have relied on highly conserved protein markers that evolve in as “clock-like” a manner as possible: e.g., ribosomal proteins. Similarly, conserved housekeeping genes that are universally essential, such as aromatic amino acid biosynthesis genes, may also function as conserved phylogenetic markers, and given their unique gene duplication history and recruitment into the scytonemin gene cluster, this provides the basis for an interesting case study to use core aromatic amino acid biosynthetic enzymes to date key events in cyanobacterial evolution. Hence, the unique nature of the gene duplication event provides an ideal data set to estimate the timing of the origin of the scytonemin biosynthesis. Garcia-Pichel et al. use these markers to estimate the minimum age of the scytonemin operon to ∼2.1 Ga, consistent with the hypothesis that sunscreens were required after the rise of oxygen in our atmosphere and may have aided in the early rise of specific lineages within crown group cyanobacteria. The timing of this fits well with the rationale that scytonemin and microbial sunscreens would be necessary after the eventual rise of oxygen in Earth’s atmosphere in order to shield ancient cyanobacteria from UVA via passive photoprotective means.

The authors expand upon the use of their data set to date the age of cyanobacteria, estimating that the most recent common ancestor of the phylum existed ∼3.6 Ga. It is important to note that several previous studies have used concatenated protein data sets to address this question (3, 7, 8); nonetheless, the use of single marker aromatic amino acid biosynthetic proteins provides a disparate data set to carry out such molecular clock analyses. It cannot be understated how challenging it is to study events that occurred billions of years ago. As such, many molecular clock studies have attempted to address phylogenetic noise by concatenating slowly evolving proteins that are universally conserved and have low frequencies of horizontal gene transfer. Moreover, given the antiquity of these events in microbial evolution, it is important to be cautious of the challenges associated with assigning microfossils to extant lineages, as there are few definitive morphological traits in microfossils and microbial morphological convergences are widespread. Thus, the 3.6-Ga estimation for crown group cyanobacteria inherently implies that stem group cyanobacteria existed before this, most likely extending into the Late Heavy Bombardment 4.1 to 3.8 Ga. Such a scenario highlights some very open questions, but also underscores intrinsic challenges with molecular clock studies, given the uncertainty inherent in many fossil constraints and the phylogenetic markers utilized. Nonetheless, studies that characterize the impact of specific phylogenetic markers or more definitively describe the authenticity of specific fossils will in the long run increase the robustness and accuracy of future molecule clock studies.

Arguably, the dates presented by Garcia-Pichel et al. are not the most important element of the study. Rather, the synthesis of prior genetic and biochemical studies to generate hypotheses concerning the origins of cyanobacterial sunscreens is a refreshing take on how phylogenetic and molecular clock studies need to be expanded to incorporate such experimental studies. Garcia-Pichel et al. describe one example of the fascinating biological ramifications of the rise of oxygen—specifically how it may have played a role in evolving new biosynthetic pathways that undoubtedly contributed to the microbial fitness of early cyanobacteria. The authors cleverly use the rise of oxygen as a singularity in the geological record to test hypotheses concerning other facets of life, i.e., the evolution of scytonemin biosynthesis, which will hopefully spur new and novel ways to utilize molecular phylogenetics and molecular clocks to investigate deep evolutionary questions. Some may find it tempting to utilize the estimates from this study to date key events in cyanobacterial history; however, it is important to note that there will always be debates on the fossils utilized and/or controversy surrounding the prior assumptions made. Beyond the estimated dates reported in this study, there is much more to be learned and gained from how Garcia-Pichel et al. utilize a foundation of molecular biology and biochemistry to drive how molecular clocks can be used to probe geobiological questions.
